# A comparative study of deep learning for cortical lesion MRI segmentation with explainability analysis in multiple sclerosis

**DOI:** 10.1016/j.nicl.2026.104007

**Published:** 2026-05-23

**Authors:** Nataliia Molchanova, Alessandro Cagol, Mario Ocampo–Pineda, Po–Jui Lu, Matthias Weigel, Xinjie Chen, Erin S. Beck, Charidimos Tsagkas, Daniel S. Reich, Colin Vanden Bulcke, Anna Stölting, Serena Borrelli, Pietro Maggi, Sebastian Baez Lugo, Delphine Ribes Lemay, Adrien Depeursinge, Cristina Granziera, Henning Müller, Pedro M. Gordaliza, Meritxell Bach Cuadra

**Affiliations:** a Faculty of Biology and Medicine, University of Lausanne (UNIL), Lausanne, Switzerland; b Radiology Department, Lausanne University Hospital (CHUV), Lausanne, Switzerland; c MedGIFT, Institute of Informatics, School of Management, HES–SO Valais–Wallis University of Applied Sciences and Arts Western Switzerland, Sierre, Switzerland; d CIBM Center for Biomedical Imaging, Lausanne, Switzerland; e Department of Radiology and Medical Informatics, University of Geneva, Geneva, Switzerland; f Translational Imaging in Neurology (ThINK) Basel, Department of Medicine and Biomedical Engineering, University Hospital Basel and University of Basel, Basel, Switzerland; g Multiple Sclerosis Center, Department of Neurology, University Hospital Basel, Basel, Switzerland; h Research Center for Clinical Neuroimmunology and Neuroscience Basel (RC2NB), University Hospital Basel and University of Basel, Basel, Switzerland; i Division of Radiological Physics, Department of Radiology, University Hospital Basel, Basel, Switzerland; j Dipartimento di Scienze della Salute, Università degli Studi di Genova, Genova, Italy; k Department of Neurology, Icahn School of Medicine at Mount Sinai, New York City, USA; l Translational Neuroradiology Section, National Institute of Neurological Disorders and Stroke, National Institutes of Health, Bethesda, USA; m Neuroinflammation Imaging Lab (NIL), Université catholique de Louvain, Brussels, Belgium; n Department of Neurology, Hôpital Erasme, Hôpital Universitaire de Bruxelles, Université libre de Bruxelles, Brussels, Belgium; o EPFL+ECAL Lab, École polytechnique fédérale de Lausanne (EPFL), Lausanne, Switzerland

**Keywords:** Multiple sclerosis, Cortical lesions, Segmentation, Detection, Magnetic resonance imaging, Brain, Deep learning, Trustworthy AI

## Abstract

Cortical lesions (CLs) have emerged as valuable biomarkers in multiple sclerosis (MS), offering high diagnostic specificity and prognostic relevance. However, their routine clinical integration remains limited due to subtle magnetic resonance imaging (MRI) appearance, challenges in expert annotation, and a lack of standardized automated methods. We present a multi-centric comparative study of CL detection and segmentation in MRI. A total of 656 MRI scans, including clinical trial and research data from four institutions, were acquired at 3T and 7T using MP2RAGE and MPRAGE sequences with expert-consensus annotations. We rely on the self-configuring nnU-Net framework, designed for medical imaging segmentation, and propose adaptations tailored to the improved CL detection. We evaluated model generalization through out-of-distribution testing, demonstrating promising lesion detection capabilities with an F1-score of 0.64 and 0.5 in and out of the domain, respectively. We also analyze internal model features and model errors for a better understanding of AI decision-making. Our study examines how data variability, lesion ambiguity, and protocol differences impact model performance, offering future recommendations to address these barriers to clinical adoption. Furthermore, we designed and implemented a medical expert questionnaire for better assessment of clinical value of the model predictions. To reinforce the reproducibility, the implementation and models will be publicly accessible and ready to use at GitHub and Zenodo.

## Highlights

• nnU-Net enables promising cortical lesion segmentation across a large multi-center dataset.
• Comparative study evaluates nnU-Net configurations; improvements are modest and mostly non-significant.
• Expert review validates clinical relevance of false-positive predicted cortical lesions.
• Bottleneck feature analysis explores U-Net generalization limits across clinical centers.
• Study highlights practical barriers hindering clinical integration of CL segmentation.

## 1 Introduction

**Fig. 1:**
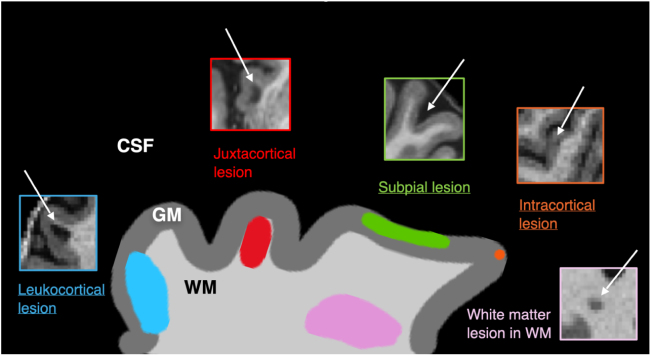
Types of MS lesions categorized by their location with respect to the cortex. Lesions appear on MP2RAGE MRI as hypointense regions (white arrow pointers). CL types are $\underline{\mathrm{underlined}}$, the rest are WMLs. Leukocortical (CL) and juxtacortical (WML) are sometimes difficult to distinguish due to unclear cortical involvement. CSF— erebrospinal fluid, GM—gray matter, WM—white matter.

Multiple sclerosis (MS) is a chronic inflammatory and neurodegenerative disease of the central nervous system, affecting over 2 million people worldwide (Reich et al., 2018, Portaccio et al., 2024). Magnetic resonance imaging (MRI) is central to the diagnosis and monitoring of MS, enabling the detection of characteristic brain lesions (Hemond and Bakshi, 2018, Filippi et al., 2019, Wattjes et al., 2021, Kolb et al., 2022). White matter lesions (WMLs) have served as the primary radiological hallmark, guiding diagnosis and treatment decisions (Hemond and Bakshi, 2018). In recent years, additional imaging biomarkers – such as cortical lesions (CLs) (Beck et al., 2022, Cagol et al., 2024), paramagnetic rim lesions (Absinta et al., 2019, Maggi et al., 2020), and the central vein sign (Sati et al., 2016, Cagol et al., 2024) – have gained recognition for their ability to support the diagnosis or to capture disease activity more comprehensively. CLs are of clinical interest due to their association with cognitive impairment, progressive disability, and early-stage onset (Beck et al., 2022, Beck et al., 2024, Cagol et al., 2024). Indeed, CLs have diagnostic value and were incorporated in the 2017 revision of the *McDonald criteria* due to their high specificity for MS (Thompson et al., 2018). Unlike WMLs that appear in numerous neuroinflammatory and neurovascular conditions, the presence of a CLs enhances diagnostic certainty for MS and helps differentiate it from clinical conditions mimicking MS (Thompson et al., 2018, Cagol et al., 2024). This distinction is crucial for preventing misdiagnosis and inappropriate treatment, particularly as the McDonald criteria generally favor sensitivity over specificity. Yet, despite their clinical relevance, CLs (as distinct from juxtacortical lesions) remain underused in routine practice due to several technical barriers: their small size (e.g., several mm intracortical lesions), low contrast on standard FLAIR or T1w MRI sequences, and the need for expert radiological interpretation (Kolb et al., 2022). Different MS lesion types are illustrated in Fig. 1.

Automated segmentation methods offer a promising avenue to facilitate the adoption of CL assessment into clinical workflows. They can reduce the burden of manual annotation, increase reproducibility, and enable broader access to quantitative lesion metrics. However, the development and deployment of such tools are hindered by several caveats: limited annotated datasets, scanner and protocol variability, and poor generalization across sites. CL segmentation, in particular, is further challenged by *high class imbalance* and substantial heterogeneity in lesion appearance. These limitations have slowed the translation of automated CL assessment tools into clinical use.

Deep learning (DL) approaches for CL segmentation have been limited by the scarcity of adequately annotated training data. Consequently, initial DL approaches employed joint segmentation strategies, where CLs and WMLs were treated as a single lesion class without distinction. Concretely, in F.L. Rosa et al. (2020), authors proposed a shallow 3D U-Net model (Ronneberger et al., 2015, Çiçek et al., 2016) for this joint segmentation using fluid-attenuated inversion recovery (FLAIR) and magnetization prepared 2 rapid gradient echo (MP2RAGE) (Kober et al., 2012) sequences. While this approach reported improved detection compared to previous non-DL methods (Fartaria et al., 2019), the joint classification inherently inflated CL detection performance, as predictions in juxtacortical white matter regions – which would constitute false positives in CL-specific segmentation – were correctly classified as WMLs, thereby avoiding penalization despite anatomical imprecision.

Dedicated CL segmentation with DL was first explored using ultra-high-field (7T) MRI, which enhances the visibility of CLs (Beck et al., 2018). The CLAIMS framework (Rosa et al., 2022a) demonstrated substantial improvements in CL-specific detection, particularly for leukocortical and subpial lesions. Crucially, this approach required explicit CL identification, meaning any detections in white matter regions (including juxtacortical areas) were counted as false positives rather than alternative lesion types. When accounting for this methodological difference, the CL detection performance was comparable to previous joint segmentation approaches, but with significantly improved specificity for cortical tissue. Despite its effectiveness, the application remains limited due to the low availability of 7T scanners in typical clinical settings and modest inter-rater reliability even at these high fields.

Further advances aimed at leveraging enhanced contrast visibility on conventional scanners include combining multiple advanced contrasts or synthetic sequences. Gordaliza et al. (2025) investigated the impact of fluid and white matter suppression (FLAWS) alongside MP2RAGE using the 3D nnU-Net (Isensee et al., 2021) framework. This CL-specific approach at 3T achieved detection results comparable to earlier methods while maintaining the anatomical precision required for clinical CL assessment. However, its clinical translation is hindered by the requirement for FLAWS sequences not routinely implemented in clinical protocols, potentially limiting widespread adoption. Similarly, Dwyer et al. (2025) evaluated retrospective segmentation methods leveraging legacy clinical trial data, introducing novel contrast combinations such as FLAIR${}^{2}$, T1/T2 ratio, and AI-derived synthetic double inversion recovery (DIR) contrasts. Their findings highlighted improved CL detection when using synthetic MRI-derived contrast maps and emphasized the persistent challenges related to variability across scanner platforms and clinical settings.

While these studies demonstrate significant progress in automated CLs segmentation, a comprehensive evaluation of different DL architectures and their comparative performance remains unexplored. Most works have focused on optimal input contrast combinations, leaving questions about model architecture selection, generalizability across clinical settings, and failure case analysis largely unaddressed.

Furthermore, clinical deployment of DL models requires robust performance across diverse imaging protocols, scanner vendors, and field strengths that differ significantly from controlled research environments. Previous generalizability assessments have been limited in scope, with studies testing on relatively small out-of-domain cohorts (36 subjects in F.L. Rosa et al. (2020), 20 subjects in Rosa et al. (2022a)) using similar scanner types and protocols. Such evaluations may not adequately reflect the heterogeneity encountered in real-world clinical settings, where scanner vendors, acquisition parameters, and patient populations vary substantially. Additionally, the complex nature of CL detection, where subtle anatomical distinctions determine diagnostic relevance, necessitates comprehensive error analysis to understand model behavior and failure modes—an aspect that has not been systematically addressed in prior CL segmentation literature.

## 2 Contributions

We present a multi-centric comparative study of CL segmentation using a large multi-center dataset comprising 656 scans from 4 medical centers across different field strengths (3T/7T) and acquisition protocols. Our framework leverages commonly acquired MP2RAGE and MPRAGE sequences to ensure clinical applicability. Specifically, our contributions include:

• A systematic architectural comparison identifying effective model configurations for CL segmentation across diverse clinical settings,
• Out-of-distribution evaluation on 224 subjects across different scanner vendors, acquisition protocols, and patient cohorts—one of the most heterogeneous OOD assessments in CL segmentation to date,
• Systematic error analysis in CL segmentation, identifying failure modes and providing actionable insights for model improvement and clinical interpretation,
• Public release of model weights and implementation framework to enable widespread adoption in clinical and research environments.

## 3 Materials and methods

### 3.1 Data

Four medical centers shared their longitudinal and cross-sectional data for this study, including (A) Basel University Hospital (USB), Switzerland, (B) University Hospital of Lausanne (CHUV), Switzerland, (C) National Institutes of Health, USA, and (D) Catholic University of Louvain (UCLouvain), Belgium. The data information is provided in Table 1.

#### 3.1.1 Manual annotation

The scans were manually annotated using local resources and annotation guidelines. In sites A–D, all lesions involving the cortex were included in the CL masks, including intracortical, leukocortical, or subpial, typically extending for at least 3 mm in the longest diameter. In A, CLs were segmented on 1 mm isotropic MP2RAGE through an independent assessment of two raters (neurologist and MD with five years of neuroimaging experience). In B, CLs were detected using $1\times 1\times 1.2\;{\mathrm{mm}}^{3}$ 3D FLAIR, 3D DIR, and MP2RAGE acquired at 3T by two independent raters (an experienced neurologist and radiologist), followed by a consensus review; then, a trained technician manually delineated the consensus lesions. In C, CLs were identified and segmented on 3 and 7T images independently. 7T visible CLs were segmented on 0.5 mm isotropic MP2RAGE (median of four acquisitions in the same scanning sessions) and 0.5 mm multi-echo T2*w GRE by two independent raters (neurologists with two and > 10 years of MS neuroimaging experience), followed by a consensus review. 3T CLs were independently segmented on 1 mm isotropic 3D FLAIR and MP2RAGE by two raters (neurologists with two and four years of MS neuroimaging experience), followed by a consensus review. In D, the detection of CLs was performed using 0.7 mm isotropic 3D DIR and MP2RAGE by three independent raters (a neuroscientist and two neurologists with three, five, and 10 years of MS neuroimaging experience, respectively), followed by a consensus review; the trained neuroscientist rater performed the manual delineation of consensus lesions.

All scans from site B and 163 scans from site A (the first time point) had information about the lesions subtypes (either from MP2RAGE or FLAIR; see Fig. 1) and included FLAIR-based WML segmentation masks. These WML masks were used exclusively for post-hoc error characterization (analysis of overlaps between model errors and WML regions), and were not included in model training or evaluation. Subjects from site C 7T data had information about the lesion types and segmentation of *potential lesions*. Potential lesions were the ones where expert raters could not conclude whether lesions were CL or not, and were not used during training or evaluation. The rest of the test data did not contain WML masks or lesion subtypes.

**Table 1:** Data description. RR—relapsing-remitting, PP—primary-progressive, SP—secondary-progressive, CIS—clinically isolated syndrome, Q2—second quartile, IQR—interquartile range.

Medical center	A	B	C	D
Demographics				

# patients	163	43	35	112
Phenotype	RR (97), PP (22), SP (44)	RR	RR (24), PP (1), SP (9)	CIS (3), RR (44), PP (8), SP (17), MS-mimic (40)
% female	60%	58%	63%	70%
EDSS Q2 (IQR)	3 (1.5-4.5)	1.5 (1.5-4)	2.5 (1-4)	2.5 (2-4.5)
Timepoints	2	2 (2 year distance)	3	1

MRI				

Scanner	Magnetom Prisma, Siemens Healthineers	Magnetom Trio, Siemens Healthineers	3T Skyra and 7T whole-body research system, Siemens Healthcare	SIGNA™, GE Health Care
Field strength	3T	3T	3T, 7T	3T
MPRAGE resolution, acquisition parameters (TR/TE/TI, ms)	–	1.0×1.0×1.2, 2300/2.84/900	–	1×1×1, 2186/3/900
MP2RAGE resolution, acquisition parameters (TR/TE/TI1/TI2, ms)	1.0×1.0×1.0, 5000/2.98/700/2500	1.0×1.0×1.2, 5000/2.84/700/2500	for 3T: 0.8×0.8×0.8, 5000/2.9/700/2500, for 7T: 0.5×0.5×0.5, 6000/5/800/2700	1×1×1, 5000/3/700/2500

Processing				

Registration	–	–	–	Registration to 3D EPI with 0.67 × 0.67 × 0.67 mm
Skull-stripping	HD-BET (Isensee et al., 2019) to FLAIR + morphological dilation (2 iterations, 3 × 3 × 3 structural element)		SynthSeg (Billot et al., 2023) to MP2RAGE + manual correction for 7T	HD-BET to MPRAGE + morphological dilation

#### 3.1.2 Data splitting

The data was split into training (Train), in-domain pure testing (Test-in), and out-of-domain (OOD) testing (Test-out). The data from site D was used for the OOD testing, due to the difference in the scanner manufacturer, compared to the other centers. The medical center possesses the clinically used MPRAGE modality (in addition to the DIR and MMP2RAGE used for lesion annotation) and data for both MS and clinical conditions mimicking MS (MS-mimic) patients. Thus, we can test the applicability of the proposed framework to support MS differential diagnosis. The rest of the data A–C were separated on Train and Test-in by using a custom stratified split approach that preserved both subject-level grouping and data distribution characteristics. The method first aggregated data at the subject-site level and then applied stratification techniques while ensuring subjects from the same site remained together in the same split. The implementation maintained similar distributions of site, lesion count, and total lesion volume between splits by creating composite stratification categories. The continuous variables (number of lesions and total volume) were discretized into 5 quantile-based bins, and single-sample stratification groups were merged with their nearest neighbors based on similarity in lesion metrics while respecting site constraints. This approach allocated 80% of the in-domain data to training and 20% to testing, with reproducibility ensured through fixed random seeding. Distribution statistics were verified through a comprehensive comparative analysis that examined the percentage distributions of categorical variables across splits, assessed descriptive statistics (mean, standard deviation, quartiles) of the continuous variables in each partition, and confirmed the preservation of subject-site integrity by counting unique subject-site combinations in the resulting datasets. Table 2 suummarizes the results of data splitting.

**Fig. 2:**
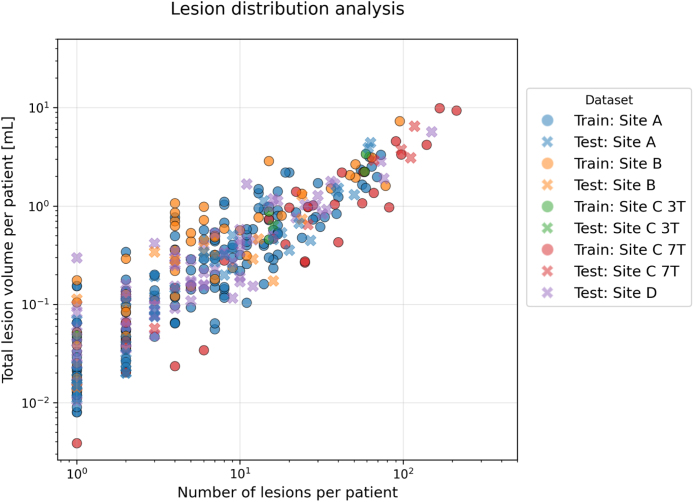
Distribution of total lesion volume in milliliters and number of lesions per patient across different medical sites.

The preprocessing pipelines differ across sites, reflecting the practical constraints of a real-world multi-center study: data sharing agreements, local ethics requirements, and anonymization procedures vary by institution, meaning that available sequences and pre-applied processing steps differ. Where skull-stripping was not already applied, we used the tool most appropriate for each acquisition type: HD-BET (Isensee et al., 2019) for standard 3T data at sites A, B, and D, and SynthSeg (Billot et al., 2023) for the 7T MP2RAGE data at site C, where the intensity distribution differs substantially from 3T. Site D additionally required registration to a 3D EPI reference space to co-register its multi-contrast acquisitions. Overall, the differences between A–D sites are driven by varying acquisition and annotation protocols, as well as patient cohorts. The differences in the distributions of lesions across different sites and domain are shown in Fig. 2. Sites A, B, and C have patients with different total lesion volumes and numbers of lesions per patient; however, this distribution is skewed towards smaller lesion volumes and counts. Site C, containing mostly ultra-high-field 7T data, has higher total lesion volumes and counts per patient since the visibility of CLs on 7T increased sensitivity for CL detection (Madsen et al., 2021). Moreover, the annotations performed on 7T MP2RAGE and T2-star-weighted (T2*w) GRE images resulted in the detection of many subpial lesions.

**Table 2:** Details of the data split. Modality 1 - MPRAGE, 2 - MP2RAGE.

Set name	Partition			Site (Number of Scans)			
	Modality	**7T**	MS-mimic	A	B	C	D
Train	2			191	60	5	0
	2	✓		0	0	31	0
	1			0	60	0	0

Test-in	2			50	14	1	0
	2	✓		0	0	6	0
	1			0	14	0	0

Test-out	2			0	0	0	72
	2		✓	0	0	0	40
	1			0	0	0	72
	1		✓	0	0	0	40

Total				241	148	43	224

### 3.2 Comparative study

We employed the nnU-Net framework (Isensee et al., 2019, Isensee et al., 2024), a fully automated, self-configuring DL method widely regarded as a state-of-the-art approach for biomedical image segmentation tasks. While nnU-Net provides automated optimization for key segmentation pipeline components, it allows for user modifications at critical stages. nnU-Net automatically optimizes the entire segmentation pipeline, including preprocessing, U-Net network architecture configuration, training procedure, inference, and post-processing, based solely on dataset characteristics. The framework’s default process includes intensity normalization, resampling to a heuristically configured target voxel spacing (resolution adjustment), cropping or padding to standardized patch sizes, and data augmentation (e.g., rotation, scaling, and elastic deformations) to enhance robustness.

Overall, the self-adaptive nature aims at nnU-Net’s high segmentation accuracy and robust generalization across diverse clinical imaging applications. Moreover, previous work on CL segmentation used 3D U-Net variations (F.L. Rosa et al., 2020, Rosa et al., 2022a) or nnU-Net (Gordaliza et al., 2025). The default configuration of the framework employs a dynamically configured 3D U-Net architecture with an encoder–decoder structure and skip connections. Training typically uses a combined Dice and binary cross-entropy (BCE) loss function, stochastic gradient descent with momentum, and polynomial learning rate scheduling during 1000 epochs. The framework implements five-fold cross-validation, providing predictions as an ensemble of all folds. nnU-Net also offers automated post-processing that removes small isolated components below a threshold volume.

In our systematic comparative experiments, we modified several key aspects of the default nnU-Net implementation to address the specific challenges of CL segmentation, like high class imbalance and the varied sizes of instances:

#### Optimization strategy.

We substituted the default stochastic gradient descent (SGD) with the Adam optimizer (Kingma and Ba, 2014) using a learning rate of $3\times 10^{-4}$. CL segmentation presents a strong class-imbalance problem: true CL voxels constitute a tiny fraction of the image volume, resulting in sparse and weak gradient signals. SGD applies a uniform learning rate to all parameters, which causes the useful gradient signals from rare CL voxels to be dominated by the abundant background, making convergence extremely slow or unstable for this task. We confirmed this empirically: SGD-based training showed no meaningful reduction in loss across epochs. Adam’s per-parameter adaptive moment estimates address this directly by tracking gradient variance independently for each parameter, allowing larger effective steps along sparse gradient directions while dampening noisy ones. The default nnU-Net SGD configuration is calibrated for large, well-represented structures such as organs, and is not well suited to the highly imbalanced CL segmentation task. Tuning SGD momentum and schedule for this task would require an expensive grid search (training takes approximately 2 days per run). The learning rate of $3\times 10^{-4}$ is a widely adopted default for Adam across a broad range of deep learning tasks; Adam’s adaptive scaling means that values within roughly an order of magnitude perform similarly, and our experiments confirmed stable convergence at this value.

#### Network architecture.

We systematically evaluated multiple architectural variants to address the unique challenges of CL segmentation. Beyond the baseline vanilla U-Net configuration provided by nnU-Net, we tested the recently introduced residual encoder variant that enhances gradient flow through deeper network layers. This residual encoder architecture is available in three configurations (designated “M”, “L”, and “XL”) with progressively larger patch and batch sizes, allowing us to examine the trade-off between computational requirements and the ability to capture broader contextual information relevant to CL identification (Isensee et al., 2024). Furthermore, we explored the application of the U-Mamba model layers (Ma et al., 2024) to the segmentation architecture. This approach was motivated by U-Mamba’s theoretical advantages in modeling long-range spatial dependencies, which we hypothesized would better capture the relationship between lesion appearance and surrounding cortical anatomy. We tested two Mamba integration strategies: a conservative approach with Mamba layers only at the bottleneck, and a more extensive implementation with Mamba layers throughout the encoder pathway. However, both U-Mamba configurations encountered significant convergence failures during training that persisted across multiple parameter settings, and were therefore excluded from the comparative analysis.

#### Loss function.

Beyond the default combination of Dice and cross-entropy losses, we evaluated its blob loss variant (Kofler et al., 2023) to better capture the morphological characteristics of small CLs.

#### Resolution standardization.

As an experimental condition (Experiment 3), we investigated whether upsampling all images to match the native resolution of the 7T site C data (0.5 mm${}^{3}$) prior to nnU-Net preprocessing could improve lesion detection. Images were upsampled using *SimpleITK* with *b-spline* interpolation before being passed to the nnU-Net pipeline, so that its heuristic-based target resolution selection would converge on 0.5 mm${}^{3}$. The rationale was to test whether preserving the fine structural detail available in the site C data could benefit detection of subtle cortical boundaries across all sites. It is important to note that this resampling strategy was evaluated in isolation as a single experimental ablation; it was not part of the standard pipeline used in all other experiments. Resampling to 0.5 mm${}^{3}$ isotropic resolution increases the number of voxels by a factor of ∼8 compared to 1 mm${}^{3}$ isotropic data, substantially increasing GPU memory requirements and training time.

#### Postprocessing.

We found that the default postprocessing, removing small instances, is suboptimal for segmenting CL, as the small size of many CLs (e.g., a few voxels) meant that true positive lesions were being inappropriately removed.

We systematically evaluated framework modifications, including architectures, losses, and preprocessing, to quantify the improvement for CL detection on a dataset with diverse acquisition protocols and field strengths.

### 3.3 Evaluation

#### 3.3.1 Metrics

We use a standard (Rosa et al., 2022b, Gordaliza et al., 2025) evaluation pipeline, including the assessment of overall segmentation employing Dice similarity score (DSC), normalized DSC (nDSC) (Raina et al., 2023a), and lesion detection quality (F1-score, Precision, and Recall).

Let TP, FP, FN be the number of true positive, false positive, and false negative voxel predictions. Then, the segmentation quality measures are defined as follows:

$$DSC=\frac{2TP}{2TP+FP+FN},$$

$$nDSC=\frac{2TP}{2TP+\kappa \cdot FP+FN},\;\kappa =h\left(r^{-1}-1\right),$$

where h is the ratio between the positive and the negative classes in the ground truth scan segmentation, r∈(0,1) is the reference value set to the mean fraction of the positive class. The nDSC accounts for the difference in the positive class load across different scans, addressing the class-load bias in the DSC.

Let *TPL*, *FPL*, and *FNL* be the number of true-positive, false-positive, and false-negative lesions. The lesions were classified on TPL, FPL, and FNL through the connected components analysis with 26 connectivity. A predicted connected component is a TPL if it has a one-voxel overlap with the ground truth; FPL if no overlap. A ground truth connected component is an FNL if it has no overlap with the prediction. Then, the detection quality measures are defined as follows:

$$F_{1}\text{-score}=\frac{2TPL}{2TPL+FPL+FNL},$$

$$\text{Precision}=\frac{TPL}{TPL+FPL},\;\text{Recall}=\frac{TPL}{TPL+FNL}.$$

To assess the statistical significance of the differences in the medians of quality metrics for different models, two-sided paired Wilcoxon tests on ranks were used. The p-values from multiple Wilcoxon tests were corrected using a Benjamini–Hochberg procedure for controlling the false discovery rate (FDR) with a 0.05 error rate.

#### 3.3.2 Detailed model performance analysis

During the comparative study we used the aforementioned metrics to select the optimal model. We then performed a detailed analysis to explain the model’s decisions.

##### Characterization of lesion-detection errors.

Sites A, B, and C manual annotations included different lesion subtypes (leukocortical, subpial, WML, etc.). We used this information to better characterize the model errors. For this, we analyzed the overlaps between TPL, FPL, and FNL masks with labeled cortical and WM masks to obtain lesion classification. Additionally, we examined the relationship between lesion volume and errors.

##### Bottleneck features analysis.

To gain insight into the internal representations learned by the network and to assess whether the model captures clinically or technically relevant variability, we performed an analysis of internal model representations on the bottleneck features extracted from the trained segmentation model. These features, located at the network’s deepest layer, encode compressed information before upsampling and prediction. This analysis enables the exploration of the influence of data characteristics such as acquisition site, imaging modality, time point, or lesion burden on the learned feature space. Understanding these relationships can help uncover potential biases in the model and provide interpretability for its behavior across diverse data sources. Such information might be crucial given the diversity of the training data used in this study.

Specifically, we focused on the best-performing model and extracted its bottleneck features from the training dataset during inference. Since the model is an ensemble, the extracted nnU-Net bottleneck features were concatenated across folds. Then, the bottleneck tensors were five-dimensional, shaped as (batch, channel, height, width, depth), where the batch dimension corresponds to augmented versions of the same input scan generated through test-time augmentation (TTA). The corresponding bottleneck features are un-flipped along the input flip axes prior to averaging, so that all augmented versions are realigned to the canonical orientation; this mirrors the un-flip applied to the segmentation predictions. An alternative aggregation strategy would be to apply global average pooling to the features before averaging across TTA versions, yielding a fully orientation-invariant global descriptor. To obtain the required (samples, features) format for dimensionality reduction, we averaged across the batch dimension and flattened the remaining spatial and channel dimensions, yielding a compact feature vector for each input scan.

We then applied dimensionality reduction to project these feature vectors into a three-dimensional space for visualization and further analysis. Two complementary techniques were employed: principal component analysis (PCA) and uniform manifold approximation and projection (UMAP). PCA is a linear method that identifies orthogonal directions of maximal variance in the data, allowing for efficient compression while preserving global structure (Bishop, 2006). In contrast, UMAP is a nonlinear technique that constructs a low-dimensional embedding by preserving local neighborhood relationships, often capturing subtle nonlinear structures in the data (McInnes et al., 2020). The use of both methods allowed us to contrast global variance-driven patterns with local manifold structures. The dimensionality reduction models were fitted exclusively on the training data and subsequently used to project the test data into the same space. This was done primarily to understand the specifics of the trained features and analyze their relationship to the testing data.

The resulting embeddings were colored and grouped according to categorical variables (such as medical center, MRI modality, and field strength). These visualizations provided a qualitative assessment of how strongly the model’s internal representation space reflects these underlying characteristics, indicating the model’s capacity to differentiate or generalize across different domains.

##### Medical expert feedback.

For the OOD data from Site D, the model lesion predictions were reviewed by two trained annotators. One neuroscientist and one neurologist with 3 and 5 years of experience in MS neuroimaging, respectively.

The goal of this experiment was multifaceted and included testing several hypotheses:

**H1** The main objective to review FPLs and identify if they are initially missed MS lesions (either CL or WML).
**H2** Assess if MS-mimic patients have focal lesions, which can be confused with WML or CL.
**H3** Check if medical experts are less confident on FPLs and FNLs compared to TPLs and why.
**H4** Check if the perceived delineation quality differs across TPL, FPL, and FNL groups, to assess the validity of CL delineation quality metrics.

For this, we first selected all FPLs from MS patients (N = 109) predicted by the best model on MP2RAGE scans—modality used for CL annotation in Site D. Then, we sampled size-matched FPLs (n = 50) and TPLs (n = 50) from the ground truth masks. We added FPLs predicted in MS-mimic patients (n = 18). For each region of interest (ROIs), the annotators needed to review the scan with a lesion overlay using ITK-SNAP (Yushkevich et al., 2006). For each ROI, they followed the guidelines and responded to the questionnaire summarized in Table 3.

Before the experiment, an introductory session was conducted with the medical experts. The experts were provided with the written guidelines and an online table form to submit their responses. All the patient IDs were anonymized to minimize the possibility of recognition of specific patients and lesions. The experts were prohibited from consulting other contrasts.

**Table 3:** The structure of the medical expert questionnaire: questions asked for each ROI. Participants could also leave an optional comment for each ROI.

Question	Description	Test
1. ROI classification (multiple selections possible)	Classify the ROI based on the visual assessment of the specified MRI modality. If there is a chance that ROI belongs to several classes, select all secondary classes in the order of decreasing certainty. Possible classes: Intracortical MS lesion, Leukocortical MS lesion, Unclear leuko- or juxtacortical MS lesion, Juxtacortical MS lesion, Deep white matter MS lesion, Vascular lesion, Vessel, Other (custom), Unknown.	H1,H2
2. Confidence level (single selection)	Assess your confidence when selecting the ROI classification. Possible confidence levels (5-level Likert scale): Not at all confident, Slightly confident, Moderately confident, Very confident, Extremely confident.	H3
3. Reasons for decreased confidence (multiple selection possible)	Select all the reasons decreasing your confidence in the first response. Possible responses: None, Small lesion size, Unclear cortical involvement, Low image quality, Low ROI visibility , Poor segmentation quality, Other (custom).	H3
4. Delineation needs correction (yes/no)	Do you think the lesion delineation needs to be revised?	H4

## 4 Results

### 4.1 Comparative study

#### 4.1.1 Experiment 1: architectures comparison

The model performance for different architectures is shown in Fig. 3, and the values of the metrics with standard errors are shown in Table 4.

U-Mamba architectures could not finally be included in the comparative analysis as we encountered significant convergence challenges during training that prevented reliable model fitting despite multiple parameter configurations and optimization attempts. These convergence issues persisted across both the bottleneck-only and full-encoder implementations of the U-Mamba layers, aligning with known training instabilities in state-space models (Isensee et al., 2024).

Among the successfully trained architectures, the performance differences were minimal. As shown in Tables 4, the Vanilla architecture achieved the highest normalized DSC (0.499±0.038) and recall (0.632±0.038) in the *in-domain (Test-in)* dataset. At the same time, ResEncUNet variants had insignificantly fewer false-positive lesions on the *out-of-domain (Test-out)* dataset. Since the differences between the models were not significant, the choice of more computationally expensive larger architectures was not justified. Thus, we used the Vanilla architecture for further experiments.

Fig. 3Radial plots comparing different architectures using quality metric means and 90% CIs. P-values from the paired Wilcoxon statistical tests with FDR correction, comparing model X with the baseline, i.e., Default resampling (BCE + Dice Loss): * p<0.05, ** p<0.01, *** p<0.001 compared to the Vanilla baseline model.Fig. 3

**(a):**
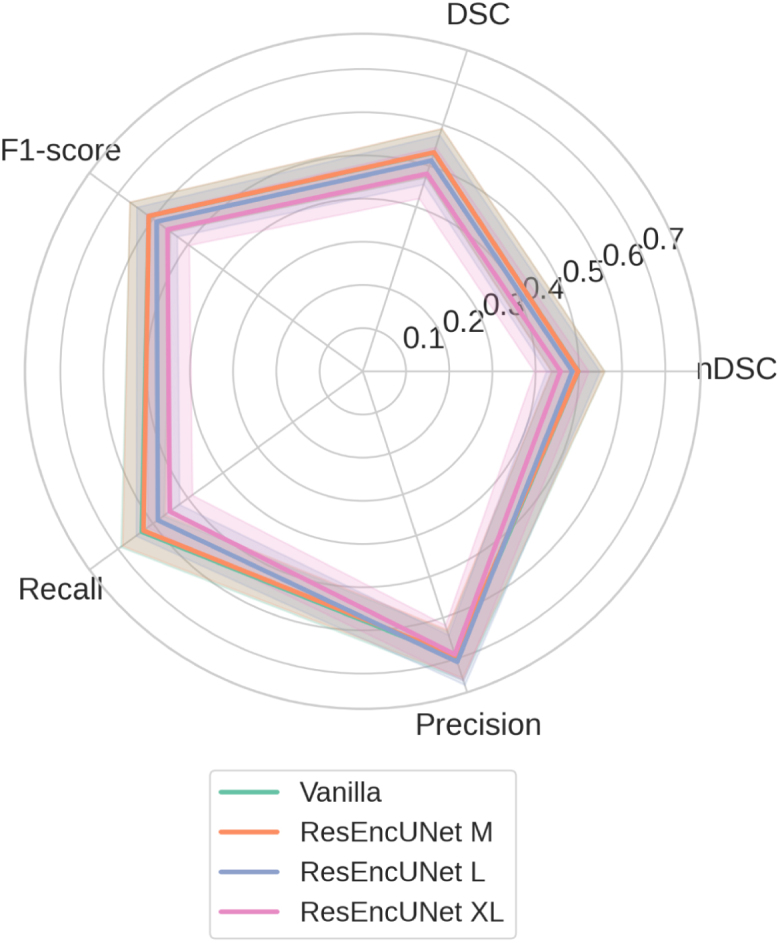
Test-in.

**(b):**
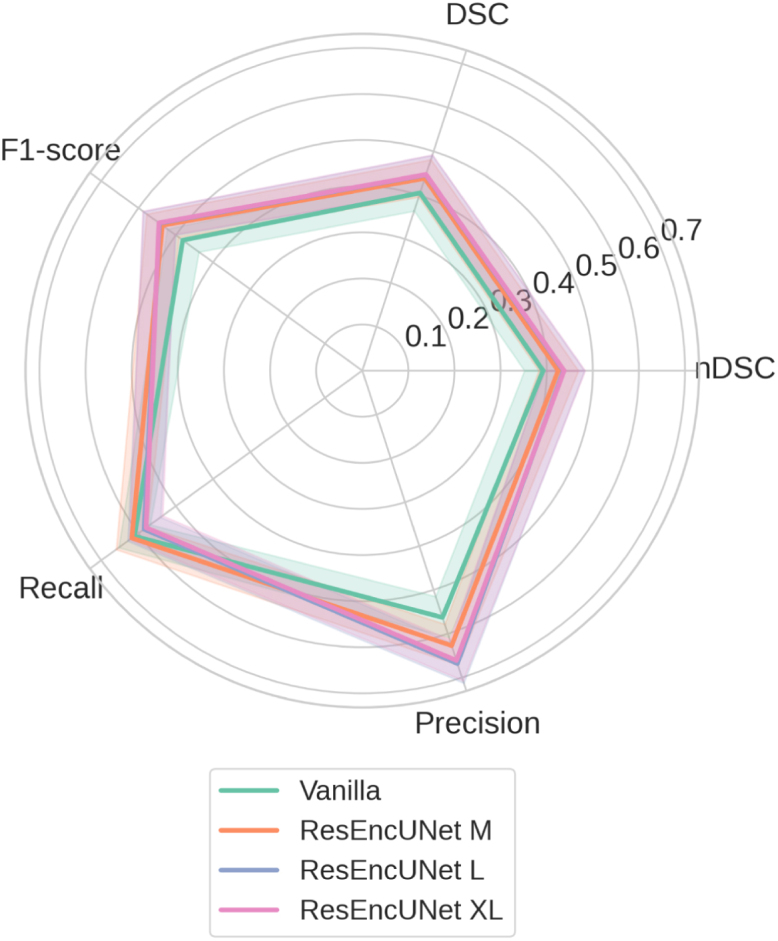
Test-out.

**Table 4:** Performance comparison of model architectures across i) Test-in and ii) Test-out domains. Values represent mean performance with standard error as subscripts. Bold values indicate the highest performance for each metric.

(a) Test-in					

Model	nDSC	DSC	F1-score	Recall	Precision

Vanilla	**0.499**_0.038_	0.532_0.036_	0.612_0.035_	**0.632**_0.038_	0.698_0.036_
ResEncUNet M	0.498_0.038_	**0.533**_0.036_	**0.612**_0.036_	0.627_0.038_	0.693_0.036_
ResEncUNet L	0.485_0.038_	0.513_0.037_	0.590_0.035_	0.586_0.038_	**0.708**_0.037_
ResEncUNet XL	0.457_0.039_	0.481_0.038_	0.559_0.038_	0.552_0.039_	0.689_0.041_

(b) Test-out					

Model	nDSC	DSC	F1-score	Recall	Precision

Vanilla	0.392_0.025_	0.405_0.025_	0.481_0.026_	0.611_0.026_	0.563_0.029_
ResEncUNet M	0.425_0.026_	0.439_0.026_	0.536_0.026_	**0.618**_0.025_	0.627_0.028_
ResEncUNet L	0.436_0.027_	0.447_0.026_	0.544_0.026_	0.585_0.026_	**0.668**_0.029_
ResEncUNet XL	**0.438**_0.027_	**0.447**_0.027_	**0.545**_0.026_	0.579_0.027_	0.661_0.028_

#### 4.1.2 Experiment 2: Blob loss effect

The Vanilla model performance for different loss functions is illustrated in Fig. 4, and the values of the metrics with standard errors are shown in Tables 5.

Blob BCE + Dice Loss showed numerically higher performance across most metrics compared to standard BCE + Dice Loss. For test-in domain, improvements included nDSC (0.505 vs 0.499), DSC (0.539 vs 0.532), F1-score (0.641 vs 0.612), and Precision (0.715 vs 0.698). Similar trends were observed out-of-domain, with the most notable gain in Precision (0.592 vs 0.563). Statistical significance was achieved for out-of-domain Precision only (p<0.05); no other metric reached significance after FDR correction.

#### 4.1.3 Experiment 3: effect of image upsampling

The model performance for different image resampling strategies is illustrated in Fig. 4, and the values of the metrics with standard errors are shown in Tables 5.

Upsampling significantly improved lesion detection, increasing Recall from 0.632 to 0.748 (p<0.001) for test-in domain. However, this came at the cost of substantially increased FPs, with Precision dropping from 0.698 to 0.296. The poor precision–recall trade-off resulted in overall performance degradation, with F1-scores decreasing by approximately 45% (from 0.641 to 0.339).

Fig. 4Radial plots comparing different losses and resampling strategies using model performance metrics means and 90% CIs. P-values from the paired Wilcoxon statistical tests with FDR correction, comparing model X with the baseline, i.e., Vanilla: * p<0.05, ** p<0.01, *** p<0.001 compared to baseline model.Fig. 4

**(a):**
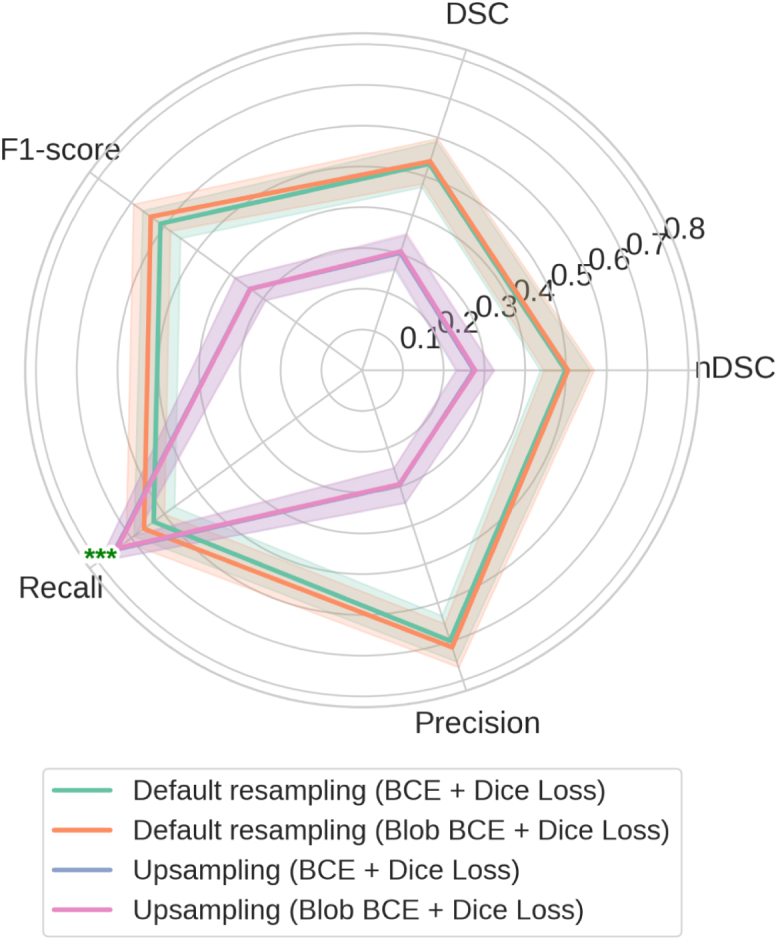
Test-in.

**(b):**
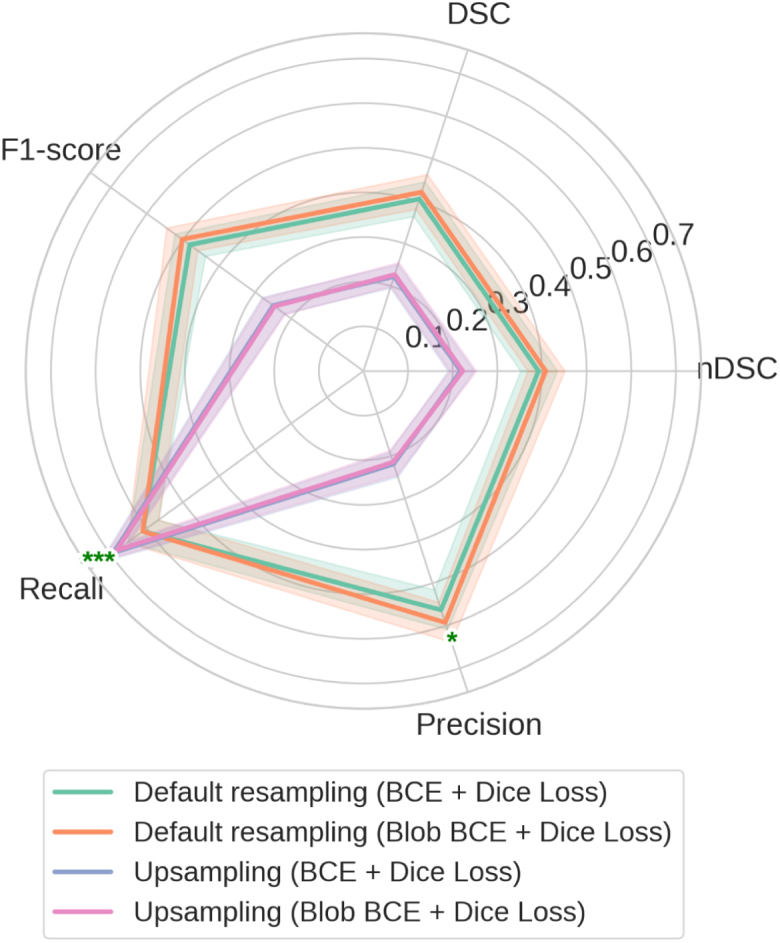
Test-out.

**Table 5:** Performance comparison of loss functions with and without upsampling across (i) Test-in and (ii) Test-out domains. Values represent mean performance with standard error as subscripts. Bold values indicate the highest performance for each metric.

(a) Test-in						

Loss function	Up-sampling	nDSC	DSC	F1-score	Recall	Precision

BCE + Dice		$0.499_{0.038}$	$0.532_{0.036}$	$0.612_{0.035}$	$0.632_{0.038}$	$0.698_{0.036}$
	✓	$0.274_{0.029}$	$0.303_{0.028}$	$0.340_{0.028}$	${\mathrm{0.748}}_{\mathrm{0.033}}$	$0.296_{0.028}$

Blob BCE + Dice		${\mathrm{0.505}}_{\mathrm{0.038}}$	${\mathrm{0.539}}_{\mathrm{0.036}}$	${\mathrm{0.641}}_{\mathrm{0.034}}$	$0.661_{0.036}$	${\mathrm{0.715}}_{\mathrm{0.034}}$
	✓	$0.277_{0.028}$	$0.307_{0.028}$	$0.339_{0.029}$	$0.743_{0.034}$	$0.294_{0.028}$

(b) Test-out						

Loss function	Up-sampling	nDSC	DSC	F1-score	Recall	Precision

BCE + Dice		$0.392_{0.025}$	$0.405_{0.025}$	$0.481_{0.026}$	$0.611_{0.026}$	$0.563_{0.029}$
	✓	$0.218_{0.017}$	$0.223_{0.017}$	$0.250_{0.019}$	${\mathrm{0.692}}_{\mathrm{0.023}}$	$0.219_{0.017}$

Blob BCE + Dice		${\mathrm{0.408}}_{\mathrm{0.026}}$	${\mathrm{0.421}}_{\mathrm{0.025}}$	${\mathrm{0.502}}_{\mathrm{0.026}}$	$0.611_{0.026}$	${\mathrm{0.592}}_{\mathrm{0.029}}$
	✓	$0.223_{0.018}$	$0.227_{0.017}$	$0.246_{0.019}$	$0.683_{0.024}$	$0.215_{0.017}$

### 4.2 Best model analysis

We selected the Vanilla nnU-net with Blob loss model for a more detailed performance evaluation and further explainability analysis.

#### 4.2.1 Details on the model performance

The model performance in terms of lesion segmentation and detection across different sites, modalities, and diseases is shown in Table 6. According to the Kruskal–Wallis H-test with FDR correction, the differences between sites and sites × modality are significant for all metrics, except for the precision: adjusted p-value = 0.384 for precision and < 0.008 for other metrics. Among the MS patients, site A has the highest DSC and detection F1. Around a 30% drop in DSC and F1 was observed for site B compared to site A. Within site B, the difference between MPRAGE and MP2RAGE is marginally small and is not significant according to the U-test (adjusted p-value = 0.877 for DSC and 0.933 for F1). An additional 50% drop in DSC and F1 was observed for the site C 7T data; however, it only had 6 testing cases. FNL should largely explain this drop in performance, since the number is outstandingly high. OOD lesion detection performance was affected by a larger number of false negatives: #FNL in sites A and B was around 3 lesions per scan, in site D, 6.5 lesions per scan. We compared the OOD site D with the in-domain sites (A, B, C) performance using the Mann–Whitney U-test with FDR correction of p-values, and detected a significant difference in the nDSC, DSC, and F1 with adjusted p-values of 0.015, 0.004, and 0.007, respectively. However, the precision, recall, and FPL and FNL counts are not significantly different.

**Table 6:** Segmentation and lesion detection quality for the best model (Vanilla nnU-Net with Blob loss) per site and modality (1 - MPRAGE, 2 - MP2RAGE; 7T if ultra-high field, else 3T). Site D has a separation on MS and MS-mimics. Mean average and standard error were computed across subjects from Test-in (A, B, C) and Test-out (D).

(a) Segmentation quality						

Site	MS-mimic	Moda-lity	FS	n	nDSC	DSC

A		2	3T	50	0.604_0.046_	0.627_0.044_

B		1	3T	14	0.445_0.090_	0.473_0.092_
		2	3T	14	0.420_0.089_	0.431_0.090_

C		2	3T	1	0.215_0.000_	0.404_0.000_
		2	7T	6	0.068_0.012_	0.233_0.059_

D		1	3T	72	0.298_0.028_	0.319_0.029_
		2	3T	72	0.292_0.030_	0.311_0.029_
	✓	1	3T	40	0.600_0.078_	0.600_0.078_
	✓	2	3T	40	0.625_0.078_	0.625_0.078_

(b) Lesion detection quality									

Site	MS-mimic	Moda-lity	FS	n	F1-score	Recall	Precision	#FPL	#FNL

A		2	3T	50	0.742_0.038_	0.783_0.039_	0.741_0.041_	1.9_0.3_	2.7_0.6_

B		1	3T	14	0.536_0.095_	0.554_0.097_	0.677_0.103_	1.1_0.4_	3.7_1.0_
		2	3T	14	0.532_0.092_	0.538_0.090_	0.679_0.107_	1.0_0.4_	3.6_0.9_

C		2	3T	1	0.593_0.000_	0.471_0.000_	0.800_0.000_	2.0_0.0_	9.0_0.0_
		2	7T	6	0.302_0.062_	0.216_0.051_	0.645_0.060_	7.0_3.1_	55.5_15.9_

D		1	3T	72	0.446_0.038_	0.395_0.038_	0.586_0.046_	1.8_0.4_	6.5_1.3_
		2	3T	72	0.435_0.037_	0.395_0.038_	0.577_0.047_	1.7_0.4_	6.7_1.4_
	✓	1	3T	40	0.600_0.078_	–	0.600_0.078_	1.5_0.7_	–
	✓	2	3T	40	0.625_0.078_	–	0.625_0.078_	0.8_0.3_	–

#### 4.2.2 Lesion-detection error analysis

**Fig. 5:**
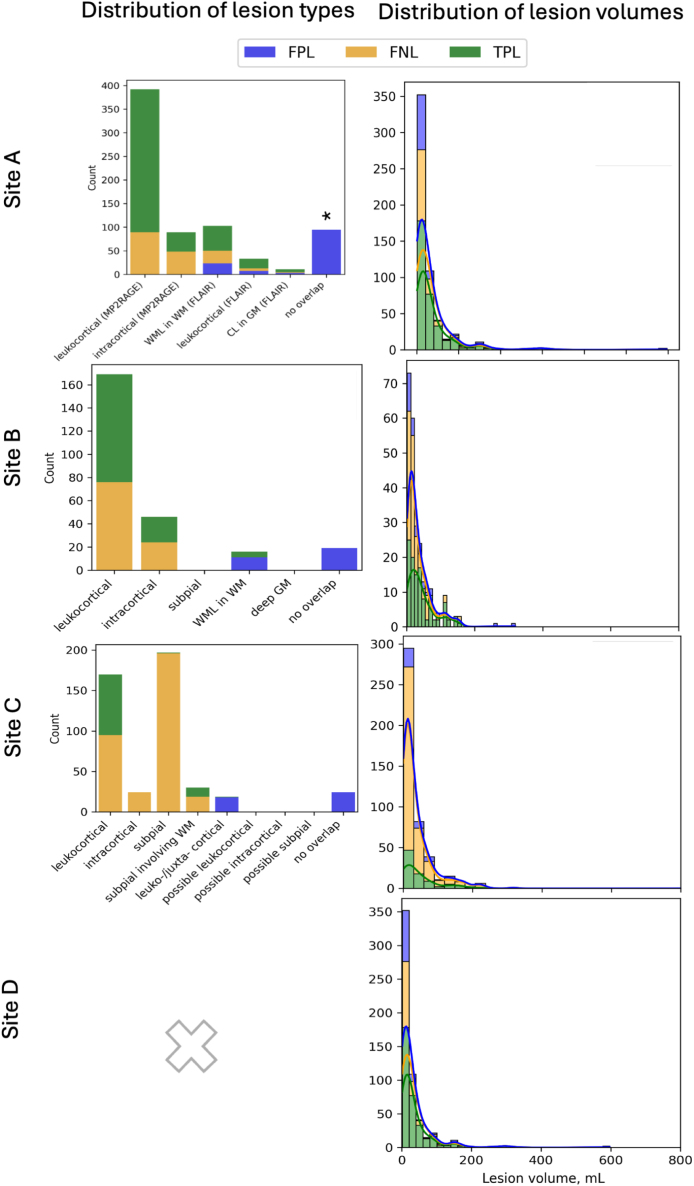
Distributions of lesion types and volumes across FPL, FNL, and TPL categories from sites A-D (rows). ${}^{⋆}$For site A WML segmentation was only available for timepoint 1, limiting FPL typization.

The distribution of the lesion types and sizes across the TPL, FPL, and FNL groups is shown in Fig. 5.

For both site A and B, we observed that the leukocortical lesions are more commonly undetected, compared to other types of lesions (65% of all FNLs from site A and 75% from site B). Also, we see that some FPLs had intersections with WML masks or leuko-/intra-cortical lesions annotated on FLAIR. Particularly, for site A timepoint 1, where the FLAIR annotations were available, 27 of 83 FPLs (33%) fell into the category of WML in WM, 6 FPLs (7%) were leukocortical under FLAIR, and 2 FPLs (2%) into CL in GM under FLAIR (i.e., intracortical). For site C (7T MP2RAGE), out of 333 FNLs, 196 were subpial lesions (59% of all FNLs) and 95 were leukocortical lesions (29%). Out of 42 FPLs, 18 fall into the category of uncertain leuko-/juxta-cortical lesions. For all the sites, the majority of TPL are leukocortical lesions.

The distribution of lesion volumes communicates that the majority of missed lesions are small. Across sites A–C, the median FNL volume is 15mL, which is 6–18 mL less than the median TPL volume.

**Fig. 6:**
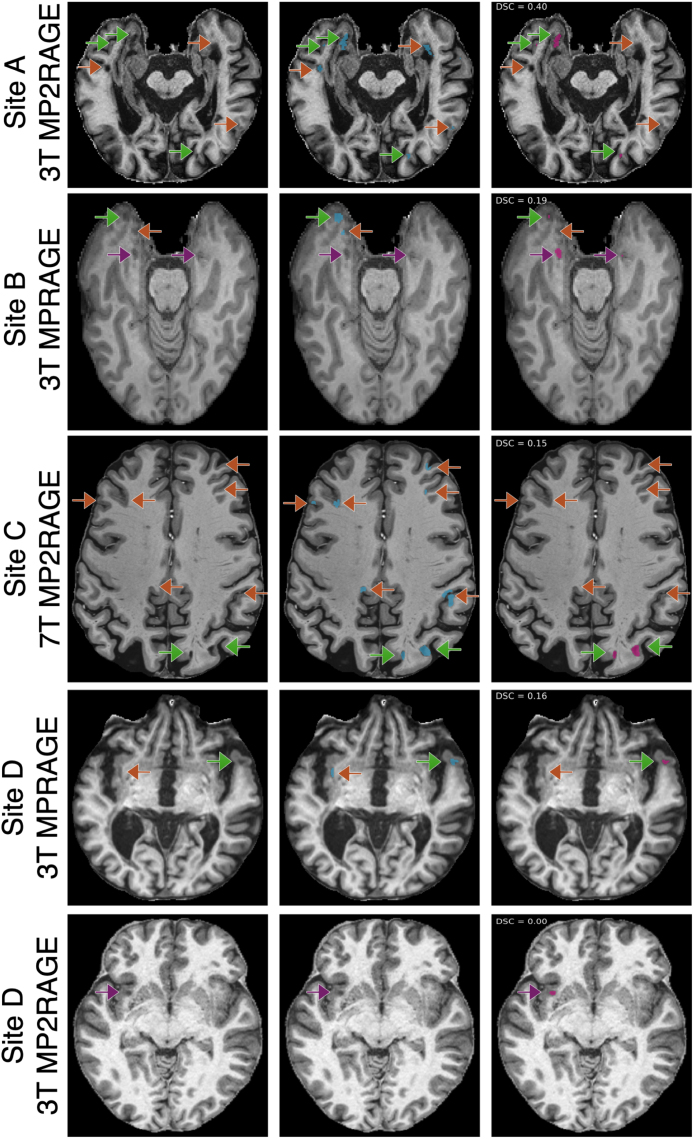
Visualization of the manual and automatic segmentation per site for scans with low segmentation quality (DSC < Q2) and median lesion count per site. Images from left to right show MRI axial slices with (i) no segmentation, (ii) ground truth mask overlay (blue), (iii) predicted mask overlay (pink).

**Fig. 7:**
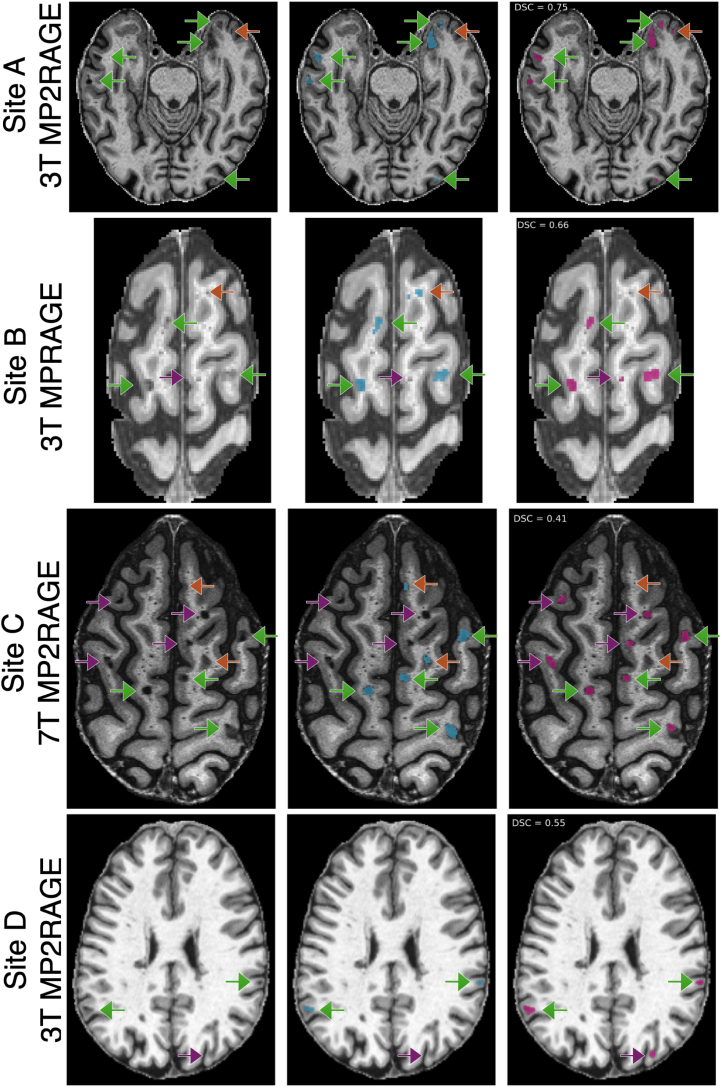
Visualization of the manual and automatic segmentation per site for scans with high segmentation quality (DSC > Q3) and median lesion count per site. Images from left to right show MRI axial slices with (i) no segmentation, (ii) ground truth mask overlay (blue), (iii) predicted mask overlay (pink).

For a qualitative assessment, we visualized subjects with low (DSC < second quartile—Q2) versus high (DSC > third quartile—Q3) segmentation quality for different sites in Fig. 6, Fig. 7.

#### 4.2.3 Bottleneck features analysis

**Fig. 8:**
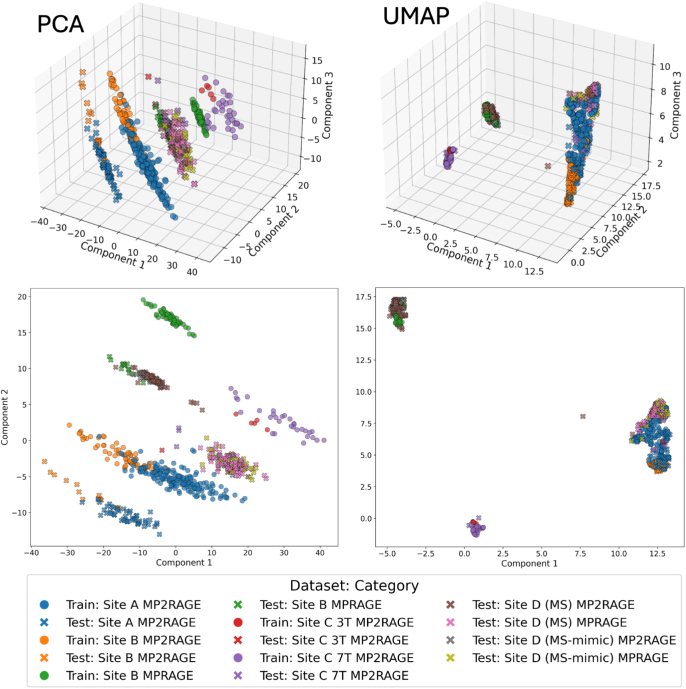
Bottleneck features visualization using PCA (left) and UMAP (right) dimensionality reduction.

The results of the nnU-Net bottleneck feature analysis are shown in Fig. 8 for PCA and UMAP methods. For PCA projection, the clusters formed partially correspond to the site × modality. The biggest cluster includes site A and site B MP2RAGE. The testing data from these sites is slightly shifted from the training data. For both PCA and UMAP, MPRAGE scans from site B and MP2RAGE scans from site C are grouped, which is due to the intensity distribution, given the relatively large distance between the MP2RAGE scans from site B of the same patients. For both PCA and UMAP, the data from site C form a distinct cluster, indicating its separation from the rest of the data. Most of the OOD test data, projected into the space of training bottleneck features, is located close to the largest cluster (sites A and B, MP2RAGE). For PCA, OOD test data has a central location for the first three components. Additional results quantifying the relationship between different continuous features, like volumetric errors, DSC, and uncertainty, with the bottleneck features are in the Appendix A.

#### 4.2.4 Medical expert feedback

The results of the medical expert feedback are presented below, following the order of our initial hypotheses (H1 to H4). The results are reported for rater 1 and rater 2, separated by a slash (“/”) by rater order.

**H1: MS lesions in FPLs.** Among MS patients, medical experts classified FPLs as MS lesions in 95.4/88.1% of cases (26.6/49.5% are CLs, 61.5/22.0% are WMLs, 7.3/16.5% are unclear CL or WML). Other selected classes include unknown objects, vessels, vascular lesions, or class combinations when unclear. Fig. 10 presents a bar plot with ROI classification by both raters.

**H2: Similarity of MS and MS-mimic lesions.** Among MS-mimic patients, 83.3/33.3% of FPLs were classified as lesions; the rest of the selected classes were vessels, vascular lesions, or a mix. Other (custom) class was selected 1/3 time(s), often suggesting sulcus presence. The inter-rater discrepancy in this category reflects differences in clinical experience with MS-mimic pathology between the two raters, combined with the small absolute number of MS-mimic FPLs (n = 18), where a few differing classifications produce a large percentage difference.

**Fig. 9:**
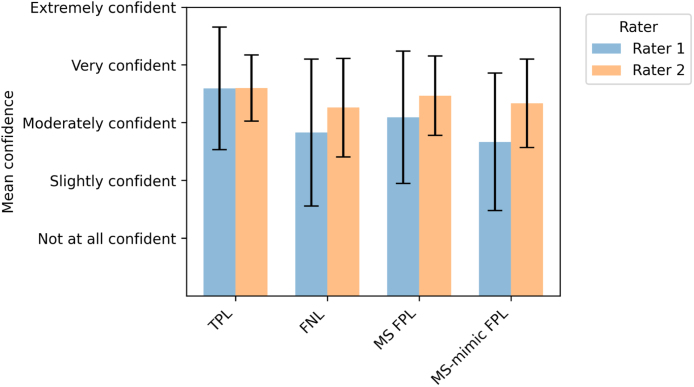
Mean and standard deviation of the confidence in ROI classification for each ROI type, provided by each rater.

**Fig. B.13:**
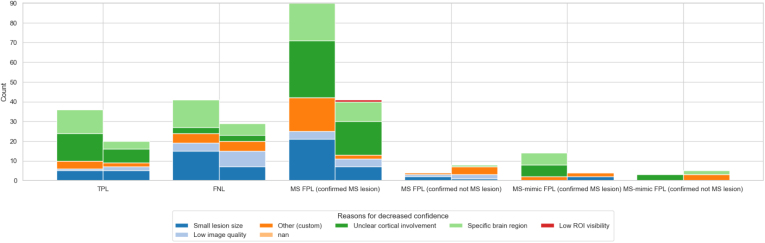
Bar plot with reasons for decreased confidence results for different lesion types provided by 2 raters (left and right).

**H3: Differences in the confidence distributions.** We compared the distribution of confidence of experts between different groups. Fig. 9 shows the mean and standard deviation of the confidence levels across ROI types. **FNLs** showed significantly lower confidence than **TPLs** (one-sided Mann–Whitney, rater 1: p = 0.0015, rater 2: p = 0.02). The Anderson–Darling (AD) test indicated a significant overall distribution difference (P-value ≈ 0.001, two-sided), suggesting that the groups diverge not only in central tendency but also in distribution shape. **MS FPLs & TPL** confidence levels are significantly different for rater 1 (P-value = 0.008), but not for rater 2 (P-value = 0.16) on a one-sided Mann–Whitney U-test; AD test P-value are 0.005 and 0.25, respectively. The reasons for decreased confidence are often attributed to small lesion size, unclear cortical involvement, and the need for an additional modality. Fig. B.13 shows a more fine-grained analysis of the reasons for decreased confidence.

**H4: delineation quality usefulness.** Rater 1 showed no statistically significant differences in the proportion of lesions needing corrections across FN, TP, MS FP, and MS-mimic FP lesion groups ($\chi^{2}$ = 5.18, P-values = 0.16), and no pairwise comparisons remained significant after FDR correction. Rater 2 has a significant overall group effect ($\chi^{2}$ = 8.09, p-value = 0.044); however, no specific group pair reached significance after multiple-testing adjustment. This global signal was primarily driven by the MS-mimic FPL group, which reported zero positive cases; however, the evidence remains weak at the pairwise level.

**Fig. 10:**
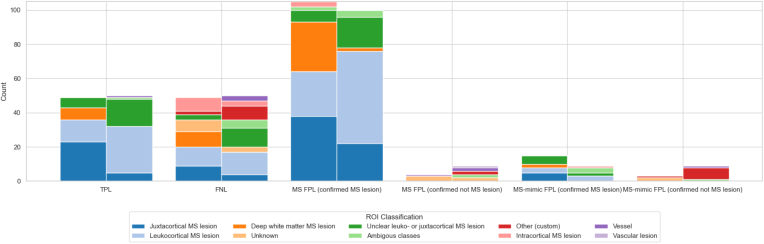
Bar plot with ROI classification results for different lesion types provided by 2 raters (left and right bars respectively in each category).

## 5 Discussion

### 5.1 Model comparison

Our experiments demonstrated that simpler neural network architectures, specifically the vanilla nnU-Net, yielded comparable or superior CL segmentation performance compared to larger architectures, which suffered from convergence difficulties. This observation aligns with prior studies advocating for lightweight models in medical image segmentation tasks. For instance, LV-UNet and Mini-Net have shown that smaller architectures can achieve competitive performance while being more efficient and easier to train (Jiang et al., 2024, Javed et al., 2024).

The customized Blob BCE + Dice Loss modestly improved precision, particularly for OOD data, reinforcing the importance of lesion-specific loss functions in optimizing segmentation tasks involving small focal lesions. A similar outcome was previously demonstrated for the WML segmentation on FLAIR MRI (Kofler et al., 2023). However, image resampling significantly improved lesion recall yet markedly increased false-positive detections, drastically reducing overall segmentation precision. B-spline MRI upsampling might lead to an increased number of artifacts, leading to higher false-positive rates. Given a promise of high recall, one could explore cascade architectures or two-step approaches to refine such segmentations with high false positive rates.

These findings collectively suggest that effective CL segmentation relies more on appropriate training strategies and task-specific loss functions than on architectural sophistication. The limited benefit of model complexity, combined with modest improvements achievable through specialized loss functions, has important implications for clinical deployment: simpler models offer substantial practical advantages, including reduced computational requirements, lower operational costs, and decreased energy consumption—critical factors for sustainable healthcare AI implementation (Topol, 2019, Topol, 2023).

### 5.2 Performance differences across sites

Substantial CL segmentation performance differences were observed across imaging sites and MRI modalities. Segmentation accuracy was highest for MP2RAGE at 3T (sites A and B; DSC 0.47–0.63, detection F1 0.53–0.73), with slightly reduced performance for MPRAGE sequences at these same sites. Conversely, ultra-high-field (7T) data from site C displayed notably poorer performance down to 0.23 DSC and 0.29 detection F1. The error analysis clarifies the nature of this failure: of 333 false-negative lesions at site C (7T), 196 (59%) were subpial lesions and 95 (29%) were leukocortical—both types predominantly visible at 7T and therefore under-represented in the training data, which consists primarily of 3T acquisitions (Maranzano et al., 2019, Beck et al., 2018). Importantly, the dominant failure mode is *missed detection of subpial lesions*, not hallucination of artifacts: precision at site C 7T was 0.645, meaning that 64.5% of model detections were correct and the false-positive count was modest (mean 7.0 FPL/scan). It is also worth noting that in clinical practice, CL assessment is primarily detection-based: the presence of even a single confirmed CL carries diagnostic relevance under the McDonald criteria (Thompson et al., 2018). Volumetric accuracy (DSC) is therefore a less clinically relevant metric for CLs than for larger structures such as WMLs, and the model’s detection capability at 7T, while imperfect, may still provide diagnostic value. For sites A-C, leukocortical lesions were frequently missed (false negatives) by the automated segmentation approach, while numerous false positives corresponded to previously annotated WMLs. This difficulty reflects inherent ambiguity in distinguishing leukocortical from juxtacortical lesions, i.e., CL and WML, a well-known challenge in manual segmentation.

Direct comparison of our results with prior CL segmentation studies is non-trivial due to differences in detection threshold (we use a 1-voxel overlap criterion), training/test cohort composition, CL definition, and whether OOD evaluation is performed. With these caveats noted, our in-domain detection F1 of 0.53–0.73 across sites A and B is broadly consistent with results reported by Rosa et al. (2022a) and Gordaliza et al. (2025) under comparable conditions. Crucially, our OOD F1 of approximately 0.44–0.50 at site D represents a substantially more challenging evaluation than in prior work, which generally tested on small cohorts with similar acquisition protocols and no true distribution shift of the scale considered here. The observed inter-rater variability in our expert feedback study is also consistent with prior reports: CL annotation is known to be more variable than WML annotation (Beck et al., 2018), reflecting subtler imaging contrast and less standardized delineation criteria. Inter-rater Dice scores for WML segmentation have been reported in the range of 0.60–0.80 (Egger et al., 2017, Carass et al., 2020), while infratentorial and spinal cord MS lesion variability is higher (Krüger et al., 2022, Gros et al., 2019, Walsh et al., 2023); CL variability likely exceeds all of these, given the additional challenge of cortical boundary determination.

### 5.3 Explainability analysis insights

The apparent clustering observed in PCA projection – where testing data from sites A and B showed shifts from training distributions – may reflect the inherent nature of learned representations or true overfitting. PCA captures only linear relationships in the high-dimensional bottleneck features, and it is expected that certain feature channels will capture scanner-specific or site-specific characteristics (acquisition noise patterns, intensity distributions, contrast variations) alongside task-relevant CL representations. This scanner-specific information naturally creates linearly separable clusters in PCA space, even when the overall learned representation remains robust for the segmentation task. Therefore, PCA alone may not be sufficient for assessing model generalization, as it cannot distinguish between harmful overfitting and expected technical variation capture. The contrasting patterns observed in UMAP projections, which preserve non-linear manifold structure, may provide more meaningful insights into the network’s actual generalization capabilities. The site- and modality-specific clustering is reduced when using UMAP projection, compared with PCA, yet it persists. This outcome underscores the critical importance of multi-center datasets and the necessity of data harmonization or domain adaptation techniques to accommodate diverse clinical contexts (Glocker et al., 2019). A centered location of the OOD data for the first three PCA components should suggest that some robust CL or T1w-modality MS brain representations were learned despite the dominant presence of some sites in the Train set.

### 5.4 Medical expert feedback on OOD data

The OOD evaluation should be indicative of the worst-case scenario model performance. Medical expert feedback provided a fine-grained analysis of the OOD performance, not reflected in the selected evaluation metrics. Particularly, we observed that up to 50% of FPLs were actual CLs, missed during the manual annotation. This finding has important implications for interpreting the quantitative metrics reported throughout this study: if up to half of the model’s apparent false positives are in fact true lesions overlooked by the original annotators, then the reported precision values systematically underestimate the model’s true performance, and F1-scores should be interpreted as lower bounds. This is consistent with the known difficulty of CL annotation, even for expert neurologists: subpial lesions in particular are inherently difficult to delineate due to their thin band-like morphology and proximity to the cortical surface, and inter-rater agreement for subpial lesions is lower than for other CL subtypes (Beck et al., 2018). Ground truth imperfection is a persistent challenge in CL research, and automated tools that can systematically flag candidate lesions may help address it by providing a structured second opinion. This shows the potential of the proposed model as a second opinion system, reinforcing medical decisions.

Some lesions are challenging for both the model and human annotators. Particularly, both raters reported FNLs to be non-MS in 40.8%–62%. The change of opinion on the ground-truth lesions and reasons for decreased confidence indicate that small lesions and lesions with unclear cortical involvement can represent a challenge for both model and human annotators. The confidence level analysis confirms that FNLs represent a significant challenge compared to TPLs for medical experts, which is not observed for FPLs vs. TPLs.

Previous discussions about the limitations of the quality measures is enhanced by this experiment results, showing that ground-truth delineation is not perfect itself. Medical experts suggest that delineation needs correction for ≈30% FPLs and TPLs.

### 5.5 Limitations and future work

An evident limitation in our study arises from the exclusive reliance on T1-weighted MRI modalities (MPRAGE and MP2RAGE). The persistent inability of models – and human observers – to reliably identify subpial lesions at 3T T1-weighted imaging confirms prior research emphasizing limited subpial lesion visibility at lower magnetic field strengths (Beck et al., 2018). Incorporating additional MRI contrasts (e.g., T2-weighted or fluid-suppressed sequences) or using ultra-high field data (Rosa et al., 2022a) might theoretically enhance segmentation quality. However, practical hurdles such as non-standardized acquisition protocols and limited availability across clinical sites significantly hinder this integration. This study again highlights that different CLs are visible on different MRI modalities, underscoring the importance of standardized lesion annotation criteria to facilitate consistent CL interpretation across studies and improve the clinical validity of automated segmentation tools.

Future studies should explore human-AI interaction systematically, assessing clinician trust in AI-generated segmentations and possible integration scenarios. Collecting feedback from the annotators about the missed and false-positive lesions would be essential for understanding the DL model decisions.

From the AI-engineering perspective, there are several future directions aiming to improve in-domain performance and generalizability. We identified that varying sizes and per-subject loads, with overall small volumes of segmented instances, still represent a challenge for the proposed model, even in-domain (Fig. 5). Potential remedies include size-dependent labeling for improved small lesion segmentation (Shang et al., 2024), inclusion of nDSC to the loss for improving fairness across subjects (Raina et al., 2023b, Spagnolo et al., 2024), or using pretrained transformer-CNN architectures (e.g., UNETR or SwinUnet) for adaptive context reasoning and semantic consistency properties. To improve the performance under the domain shift, one can adopt existing pretrained models for WML models (F.La Rosa et al., 2020) or the nnU-Net framework (Wald et al., 2025), implement synthetic lesion generation strategies (e.g., CarveMix (Zhang et al., 2023)), or consider test-time adaptation approaches (Valanarasu et al., 2024).

In clinical practice, MPRAGE and MP2RAGE are typically acquired as alternatives rather than in combination, so fusing their predictions is of limited practical scope. Nonetheless, where both are available for the same subject, averaging output probability maps could offer modest gains given their partly overlapping contrast properties. A more impactful direction would be to incorporate sequences with genuinely complementary contrast, such as T2*-weighted imaging, which provides substantially improved subpial lesion visibility. The current study does not pursue multi-sequence fusion due to data constraints, but it remains a natural direction for future work.

The aforementioned approaches would not resolve fundamental ambiguities inherent to distinguishing leukocortical from juxtacortical lesions, highlighting persistent challenges due to inherent lesion variability and high prevalence. Although quantifying exact cortical involvement remains problematic, future annotation protocols might benefit from marking uncertain lesions explicitly, enabling the use of soft labels and probabilistic approaches in training automated segmentation models.

## 6 Conclusions

This study evaluates and compares approaches to automated cortical lesion segmentation in multiple sclerosis using the nnU-Net framework. Our results show that a standard nnU-Net architecture, with task-specific adjustments like the Blob BCE + Dice Loss, effectively segments cortical lesions across different imaging centers and MRI protocols. Cross-site performance variability and the similarity between leukocortical and juxtacortical lesions remain challenging and necessitate clearer annotation guidelines, possibly involving soft labels.

Automated segmentation tools demonstrated a potential for clinical workflows as supportive diagnostic or research aids. Their role could include providing automatic lesion segmentation as a second opinion to highlight lesions potentially missed by expert raters. Additionally, the lesion delineation procedure can be significantly simplified with the presence of automatic tools, even if manual corrections are required.

## CRediT authorship contribution statement

**Nataliia Molchanova:** Writing – review & editing, Writing – original draft, Visualization, Validation, Software, Project administration, Methodology, Investigation, Formal analysis, Data curation, Conceptualization. **Alessandro Cagol:** Writing – review & editing, Validation, Resources, Data curation. **Mario Ocampo–Pineda:** Writing – review & editing, Validation, Resources, Conceptualization. **Po–Jui Lu:** Writing – review & editing, Validation, Resources, Data curation. **Matthias Weigel:** Writing – review & editing, Validation, Resources, Data curation. **Xinjie Chen:** Writing – review & editing, Validation, Resources, Data curation. **Erin S. Beck:** Writing – review & editing, Validation, Resources, Data curation. **Charidimos Tsagkas:** Writing – review & editing, Validation, Resources, Data curation. **Daniel S. Reich:** Writing – review & editing, Validation, Resources, Data curation. **Colin Vanden Bulcke:** Writing – review & editing, Validation, Resources, Data curation. **Anna Stölting:** Writing – review & editing, Validation, Resources, Investigation, Data curation. **Serena Borrelli:** Writing – review & editing, Validation, Resources, Investigation, Data curation. **Pietro Maggi:** Writing – review & editing, Validation, Resources, Data curation. **Sebastian Baez Lugo:** Writing – review & editing, Validation, Methodology, Conceptualization. **Delphine Ribes Lemay:** Writing – review & editing, Validation, Methodology, Funding acquisition, Conceptualization. **Adrien Depeursinge:** Writing – review & editing, Validation, Supervision, Project administration, Funding acquisition. **Cristina Granziera:** Writing – review & editing, Validation, Resources, Project administration, Funding acquisition, Data curation. **Henning Müller:** Writing – review & editing, Validation, Supervision, Resources, Project administration, Funding acquisition. **Pedro M. Gordaliza:** Writing – review & editing, Writing – original draft, Validation, Supervision, Software, Methodology, Investigation, Formal analysis, Data curation, Conceptualization. **Meritxell Bach Cuadra:** Writing – review & editing, Writing – original draft, Visualization, Validation, Supervision, Resources, Project administration, Investigation, Funding acquisition, Data curation, Conceptualization.

## Ethics statement

Studies involving human data were approved by the local ethics committees; informed consent was obtained from all participants before study entry.

## Figures and Tables

**Fig. A.11:**
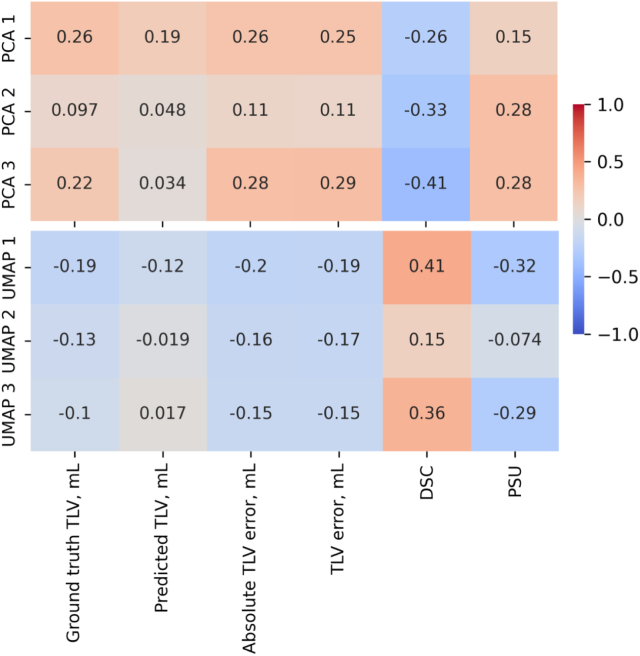
Pearson correlation between projected bottleneck features and continuous features.

**Fig. A.12:**
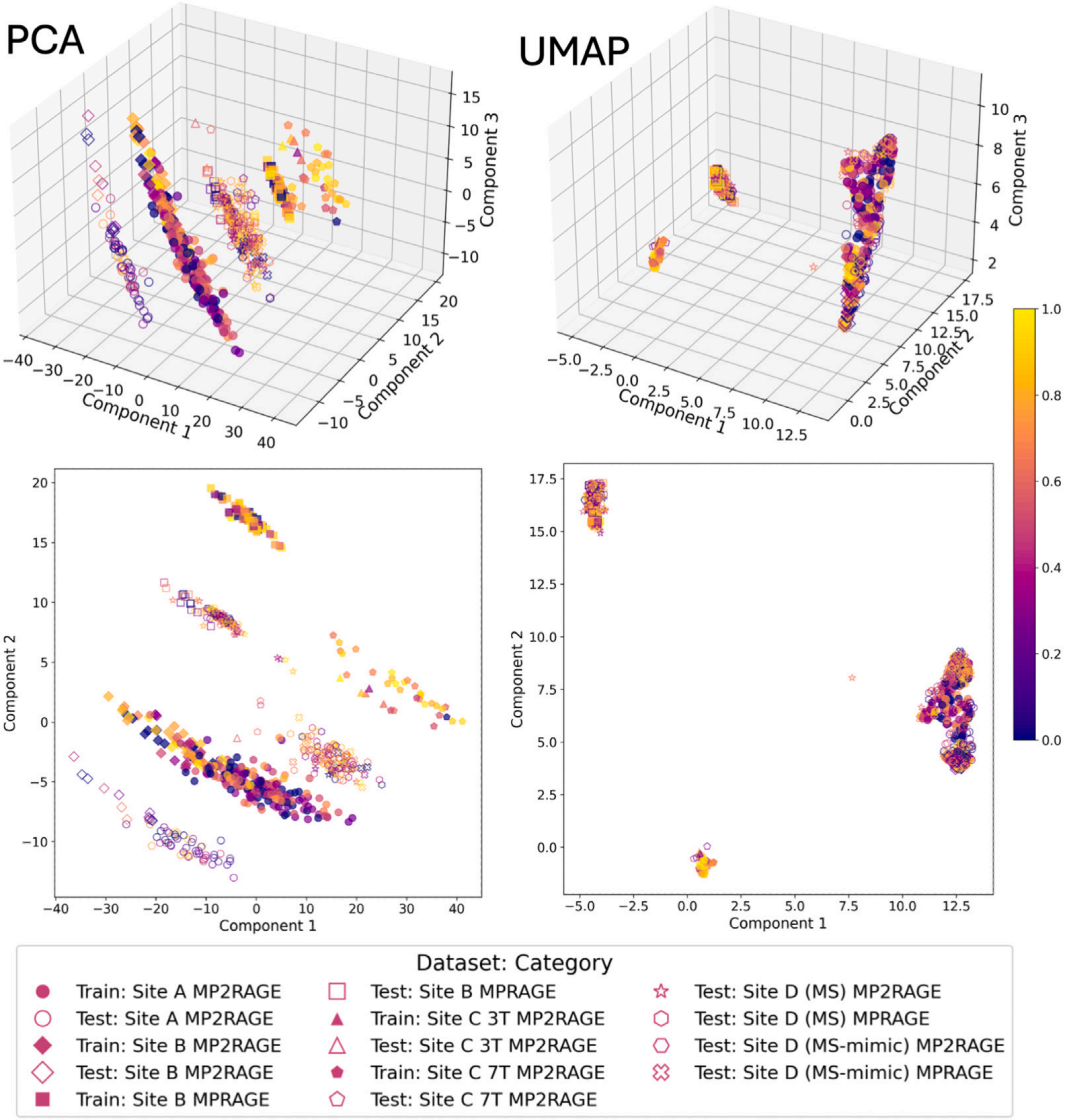
Distribution of patient-wise uncertainty ranking (computed with PSU measure) with respect to the internal model representations and sites. Latent features were projected with PCA (left) and UMAP (right). Original PSU values underwent the quantile transform to accommodate a skewed uncertainty distribution.

## Data Availability

Authors do not have rights to share the data. The code is publicly available.

## Appendix A Bottleneck space analysis

The relationship between projected internal model features and continuous features (DSC, patient-wise model uncertainty, lesion volume errors) is quantified in Fig. A.11. Mild correlation is observed between bottleneck feature components and continuous features, with the highest overall correlation values of DSC.

The patient structural uncertainty (PSU) quantifies the disagreement between individual ensemble predictions and the average ensemble prediction, reflecting both epistemic and aleatoric uncertainty (potential model errors or ambiguous data) (Molchanova et al., 2025). Fig. A.12 illustrates model features with uncertainty information. Low uncertainty examples are found across all PCA clusters of the bottleneck feature space, even in clusters distant from the Site A training set (e.g., Site B MPRAGE cluster). Conversely, Site C training and test examples consistently exhibit high uncertainty. This widespread higher uncertainty in training data across different sites may indicate significant data noise, hindering the learning of consistent representations.

## Appendix B Medical expert feedback

An additional plot regarding the reasons for the decreased confidence is given in Fig. B.13.
